# An EU-wide approach to HTA: An irrelevant development or an opportunity not to be missed?

**DOI:** 10.1007/s10198-019-01037-2

**Published:** 2019-03-14

**Authors:** Panos Kanavos, Aris Angelis, Michael Drummond

**Affiliations:** 1 Department of Health Policy & LSE Health, London School of Economics, London, UK; 2 Centre for Health Economics, University of York, York, UK

## Abstract

An EU-wide cooperation on HTA has been proposed recently by the European Commission, focusing on relative effectiveness assessment (REA) for pharmaceuticals and medical devices. This cooperation is operationalised through a proposal for a regulation. While a good step in the right direction, this HTA cooperation framework needs to be more explicit and pragmatic about clinical value definition, what constitutes quality of evidence, how real-world evidence is handled, whether the same assessment requirements will apply for medical devices as they do for pharmaceuticals, and how to safeguard consistency in REA interpretation. If demand-rather than supply-driven, this initiative can deliver wider benefits: Europe can improve its power in global drug design and development, while Member States will have at their disposal more resources to assess performance of interventions in their healthcare systems.

## Background

On January 31st, 2018, the European Commission released a proposal for a new regulation on health technology assessment (HTA) cooperation. This aims, among others, to streamline disparate national HTA processes and generate a single, joint clinical assessment, with a focus on joint relative effectiveness assessment (REA) of pharmaceuticals and certain types of medical devices, the promotion of early dialogues and the identification of emerging technologies and other opportunities for voluntary cooperation [1]. Rightfully, the scope of the draft regulation is limited to “assessment” of technologies (i.e. the scientific consideration), while “appraisal” (i.e. the decision rule) remains a national competence. The proposal has drawn on extensive impact assessment of different policy options, which, among others, found duplication in Member States’ HTA processes and argued in favour of a faster and more uniform assessment and greater equity in the availability of innovative health technologies [2]; recently, the proposal received an opinion from the European Parliament [3].

Although the draft regulation aims to provide a common foundational framework for HTA at EU level, it falls short of addressing a number of much needed changes in the assessment of medical technologies and adapting to the reality of post-clinical and post-launch data generation. Scientific challenges in novel drug discovery and development [4] and the ever-increasing use of accelerated forms of marketing authorisation by the European Medicines Agency (EMA) and other regulatory agencies provide the backdrop to the need for change by both HTAs and manufacturers.

Specifically, the HTA draft regulation needs to provide a clearer steer on what constitutes clinical value; clarify aspects relating to the quality of evidence; clarify what is the use of real-world evidence (RWE) in joint assessments and beyond; outline how should medical devices be assessed; and ensure consistency in REA interpretation.

## Clinical value definition

The credibility of a collaborative assessment at European level and its subsequent uptake by national competent authorities depend on the robustness of the process and the criteria that underpin it. In that context, it would be desirable to see a clearer definition and a more common understanding of what ‘clinical value’ is, infused with a degree of pragmatism. Beyond a technology’s clinical effectiveness and safety, dimensions such as burden of disease, severity and the level of unmet need for new treatments could be acknowledged or even accounted for. The first four of the nine domains of the European Network of Health Technology Assessment (EUnetHTA) HTA Core Model for Rapid Relative Effectiveness [5] could serve as a starting point in this discussion. It also needs to be recognised that in a number of clinical areas the traditional model of double-blind, randomised clinical trials (RCTs) may be impossible or impractical to implement. In some Member States, the mechanisms used are often too inflexible to acknowledge that. Clearer guidance is needed on additional evidence requirements that may follow the initial evidence generation. Development of common methodological guidance by Member State HTA bodies (as envisaged under the proposed regulation), could help address this; such guidance could substitute the current polyphony that exists in individual Member States about the acceptability or not of individual pieces of evidence. This transcends the issue of HTA and creates an opportunity for Europe to establish much needed infrastructure as well as a validated value framework by accounting for what is feasible or acceptable and what is not.

## Quality of evidence

A major area underpinned by significant challenges and where clear guidance is needed relates to what constitutes appropriate or acceptable quality of evidence and its assessment. A number of challenges are involved here. First, there is a divergence between the ‘political’ and the ‘patient-centric’ drive to faster approvals, increasingly through regulatory early access schemes or clinical evidence with immature data [6–8], but from an HTA perspective there is an increasing demand for evidence, more comprehensive data and more relevant comparators. While manufacturers may need to shift certain components of evidence generation to an earlier stage, this may not be possible across the board. Second, often conventional trial designs are not ethically acceptable, particularly in late stage disease when there is no standard of care, or when there is no active comparator available. Third, there is significant variability in the acceptance of comparators in different settings. By recognising this reality, EU HTA can also infuse more pragmatism in the evidentiary requirements of clinical assessment. This could mean, for example, a readiness to accept data where cohorts from RWE can compare survival in the real population versus the trial results, based on recently developed methods [9, 10]. Equally, there needs to be an acceptance of individual or mixed-treatment comparisons or indirect treatment comparisons in two- as well as single-arm trials (SATs) and a need for a general framework on when SATs are acceptable. There also needs to be recognition in evidence assessment of the heterogeneity of treatments in terms of the differences between the treatments themselves and the speed with which science is moving, for example through shorter time horizons compared with current data extrapolations over long time horizons, as well as clear signals about additional evidence generation if HTA submissions rely on immature data.

If, as its proponents suggest, the proposed regulation becomes mandatory, then there needs to be some clarity on how promising products with less mature or earlier phase data or data from non-randomised studies, will be treated from an assessment perspective. This is because the regulatory view, through EMA’s accelerated or conditional approval pathways, differs from the HTA view, which is much more conservative, considering uncertainties of evidence. If there is no clarity, then such products run the risk of a generalised ‘rejection’ on account of insufficient evidence. This, in turn, may conflict with the EC’s objective of improving the availability of innovative health technologies. There is a significant value arising from EUnetHTA in terms of facilitating a sustainable HTA collaboration in Europe and, more specifically, on methodology advances [11]. Equally, the proposed regulation has made provisions for joint scientific consultations, which, in turn, can provide clarity on evidence requirements. Still, the HTA collaboration needs to pro-actively reflect on the challenges posed by data generation and adopt a forward-looking perspective. The value of the cooperation would increase exponentially if early scientific advice could take place amongst European HTA agencies and if they all sign up to one, harmonised evidence generation base. But, unfortunately, this is unlikely to be the case; early advice initiatives identify where there may be agreements and then they identify where there are divergencies or disagreements, as for example relating to the appropriateness of comparators, among other things. Whilst early dialogue may provide some transparency, it is unlikely to expedite access to patients, unless Member States also cooperate to streamline their evidentiary requirements, possibly through such an expedited pathway for promising technologies with ‘immature’ data. The above represent areas that need to be addressed through collaborative work.

## Real-world evidence

The use of RWE is key to the proposed EU regulation. In some diseases, the value resonates in having long cohort data over many years, whereas in others, which could be driven by specific genetic subtypes, companion diagnostics and testing become very important. Obtaining these data from existing databases is usually not possible, necessitating the wider availability and use of registry data. A key challenge from an HTA perspective remains the variable acceptance of RWE. Despite improvements in infrastructure, issues remain with access to data in several Member States, including privacy issues, the lack of incentives for data sharing, availability and use, and the ongoing debate about RWE distrust. It is, therefore, fundamental to have a discussion on what needs RWE is going to fulfil. For example, while studying uncertainty in long-term effectiveness remains key, it is also important to study how the effectiveness of a new therapy can re-shape clinical practice. Having this ex ante clarity will also drive methodological and empirical developments in RWE generation further. If we accept the argument that bringing RWE into decision making will improve the quality of decisions and that expertise is needed on both sides of the data continuum, then these issues need to be addressed urgently.

## Pharmaceuticals vs. medical devices

HTA for medical devices poses significant challenges, particularly for small companies, in terms of fulfilling evidence requirements. Medical devices have a shorter life cycle than pharmaceuticals and the market is much more competitive as patents are easier to circumvent for medical devices, consequently, while long RCTs may be desirable from an evidence standpoint, they may not always be appropriate or feasible for medical devices. Unlike pharmaceuticals, medical device companies do not have data exclusivity, which means that companies who do not conduct clinical studies on their own device can often claim equivalence [12] to another device that is already on the market. Transitioning to a joint mandatory REA from a number of fragmented national and regional HTA processes, could represent a significant barrier to entry for medical device suppliers, the majority of which are small and medium-sized enterprises (SMEs).

Additionally, the number of medical devices far exceeds that of pharmaceuticals and performing HTA on all medical devices would be extremely resource intensive for all stakeholders involved even in the subset of devices envisaged by the proposed regulation (class IIb and III and subject to a scrutiny mechanism). Both assessments and re-assessments can be challenging in the medical device sector given the frequency of product modifications, the level of competition and the timing of competitor entry, which is much faster than in pharmaceuticals. Additional competitors will enter the market and have an impact on the price of an existing product, possibly placing first entrants at a disadvantage, creating an obvious barrier to entry for the first-in-class, which is absent for all followers in that class. Evidence requirements in HTA often tend to be strictest for first movers in a therapeutic area and this is likely to affect medical devices more given the market dynamics in this sector. Finally, there needs to be a common understanding about appropriate comparators for a treatment, as it is well known that countries vary substantially in defining these. The same holds for a number of therapy areas as well, such as cancer therapy, as current standard of care often differs between countries. In sum, EU HTA needs to be more circumspect about the need for REAs in medical devices and perhaps define the need, the associated evidence requirements and a methodological framework guiding these more tightly, beyond joint scientific consultations providing advice on study designs.

## Consistency in REA interpretation

A final issue relates to the consistency of REA interpretation and the ex ante definition of the rules of the game. There may be competing interests and different remits among the 80 plus EUnetHTA partner organisations from 30 countries, even among partners from the same country, which may lead to differences in assessments. Additionally, how factors such as indirect comparisons and RWE are able to provide a predictable interpretation of that evidence is important to clarify. EU-wide REAs would be beneficial so long as the reproducibility of any report and a predictable pathway to their interpretation by national competent authorities can be guaranteed. If this is not the case, then the unavoidable consequence will be further duplication of the overall HTA effort. To that end, independent assessments from a tertiary group of experts and based on common principles could minimise this.

## Overall benefits from collaborative EU HTA

The extent to which a mandatory EU-wide HTA process will reduce duplication and achieve efficiency gains depends on whether such an assessment results in (a) clarity and transparency in evidence requirements across therapeutic areas, enhanced by systematic early dialogue; (b) consistency of methods and their acceptability, recognizing the peculiarities and challenges of modern drug development; (c) clarity on the timing of performing REAs and, therefore, predictability of outcomes; and (d) a greater dialogue between the regulatory side (EMA) and HTAs around alignment of evidence requirements. This by no means implies centralisation of HTA processes; rather, common methods, joint scientific consultations and joint clinical assessments are expected to be synergistic.

There is a perception that because centralisation has been achieved for marketing authorisation, the same can be done for HTA. The two are completely different: centralisation of regulation results in a decision applicable to all, whereas HTA is not a decision, but a collection, synthesis and analysis of different information pieces that are subject to interpretation prior to arriving at a recommendation. The challenge in making this latter process mandatory is a challenge about making EU HTA demand-rather than supply-driven. If it is demand-driven and Member States use this information to shape national priorities, then there is value in generating it. But if it is supply-driven and the objective is to be churning out reports to inform the rest of Europe, then there is a danger of more duplication, unless there is buy-in from decision makers on the value of those reports. Assuming a demand-driven EU HTA, there needs to be consensus on a template for REAs; the need for such a template is already recognised by the proposed regulation.

The EU HTA cooperation is unlikely to result in substantial savings, as HTA bodies and national competent authorities will still want to understand local epidemiology and need, conduct budget impact analysis and negotiate pricing. But it can result in benefits for Member States and for Europe. Member States can free up and re-allocate resources to measure performance of interventions in the wider health care system (e.g. hospital interventions, primary care); this is urgently needed to balance the over-emphasis on drug-related HTA. From a broader perspective, this initiative would enable Europe to improve its negotiating power in global drug design and development if views can be aligned on evidence requirements. But at the moment they cannot and so the FDA and the USA still dictate to a large extent what clinical studies measure and what endpoints are used.
